# Oral Multiple Sclerosis Drugs Inhibit the *In vitro* Growth of Epsilon Toxin Producing Gut Bacterium, *Clostridium perfringens*

**DOI:** 10.3389/fcimb.2017.00011

**Published:** 2017-01-25

**Authors:** Kareem R. Rumah, Timothy K. Vartanian, Vincent A. Fischetti

**Affiliations:** ^1^Laboratory of Bacterial Pathogenesis and Immunology, Rockefeller UniversityNew York, NY, USA; ^2^The Brain and Mind Research Institute and Department of Neurology, Weill Cornell Medical CollegeNew York, NY, USA

**Keywords:** multiple sclerosis, oral therapies, anti-bacterial agents, *Clostridium perfringens*, microbiome

## Abstract

There are currently three oral medications approved for the treatment of multiple sclerosis (MS). Two of these medications, Fingolimod, and Teriflunomide, are considered to be anti-inflammatory agents, while dimethyl fumarate (DMF) is thought to trigger a robust antioxidant response, protecting vulnerable cells during an MS attack. We previously proposed that epsilon toxin from the gut bacterium, *Clostridium perfringens*, may initiate newly forming MS lesions due to its tropism for blood-brain barrier (BBB) vasculature and central nervous system myelin. Because gut microbiota will be exposed to these oral therapies prior to systemic absorption, we sought to determine if these compounds affect *C. perfringens* growth *in vitro*. Here we show that Fingolimod, Teriflunomide, and DMF indeed inhibit *C. perfringens* growth. Furthermore, several compounds similar to DMF in chemical structure, namely α, β unsaturated carbonyls, also known as Michael acceptors, inhibit *C. perfringens*. Sphingosine, a Fingolimod homolog with known antibacterial properties, proved to be a potent *C. perfringens* inhibitor with a Minimal Inhibitory Concentration similar to that of Fingolimod. These findings suggest that currently approved oral MS therapies and structurally related compounds possess antibacterial properties that may alter the gut microbiota. Moreover, inhibition of *C. perfringens* growth and resulting blockade of epsilon toxin production may contribute to the clinical efficacy of these disease-modifying drugs.

## Introduction

Multiple sclerosis (MS) is the most common non-traumatic neurological disease of young adults in Western Europe and North America (Conway and Cohen, 2010). Although traditionally considered an autoimmune disease that specifically targets central nervous system myelin (Frohman et al., 2006), increasingly, investigators have been pursuing the idea that host-pathogen interactions may play a role in the MS disease process (Collins et al., 2012). Indeed, investigations into how the gut microbiota may trigger or modulate MS relapses are currently under way. With the advent of the first oral treatments for MS, Fingolimod, Teriflunomide, and dimethyl fumarate (DMF), a reasonable question arises. Do these oral medications possess antibacterial properties, and if so, could modulation of gut bacteria contribute to protection against MS relapse?

We have previously proposed that a bacterial neurotoxin, epsilon toxin, from the anaerobic gut bacterium, *Clostridium perfringens*, may play a pivotal role in triggering newly forming MS lesions (Rumah et al., 2013, 2015; Linden et al., 2015). Epsilon toxin (ETX) is a rational candidate MS trigger due to its tropism for the blood-brain barrier (BBB) and for the myelin sheath; both of which are specifically damaged during each MS relapse (Dorca-Arévalo et al., 2008; Rumah et al., 2013; Linden et al., 2015). Remarkably, newly forming MS lesions display evidence of BBB breakdown, oligodendrocyte cell death and early microglial activation in the absence of a peripheral inflammatory infiltrate (Barnett and Prineas, 2004). While the triggering agent of these early pathologic changes remains unknown, *C. perfringens* epsilon toxin serves as a provocative candidate due to its tissue specificity and resultant mechanistic plausibility (Rumah et al., 2013, 2015; Linden et al., 2015).

*C. perfringens* is an anaerobic, spore forming, gram-positive bacillus that is sub-classified into five distinct toxinotypes based on differential exotoxin production (Table 1). *C. perfringens* type A typically colonizes the human gut with a prevalence of 63% among healthy individuals (Carman et al., 2008), while *C. perfringens* types B and D, the producers of ETX, are commonly found in the intestines of ruminant animals such as sheep, goats, and cattle but not humans (Popoff, 2011). ETX toxin is a potent neurotoxin secreted as a 33 kDa inactive precursor during the logarithmic growth phase of *C. perfringens* in the mammalian intestine. This poorly active precursor is cleaved by gut trypsin, chymotrypsin and/or an additional clostridial exotoxin, lamda toxin. The 28.6 kDa enzymatic cleavage product permeablizes the gut epithelium, enters the blood stream and binds to receptors on the luminal surface of brain endothelial cells. Once bound to brain microvessels, ETX oligomerizes and forms a heptameric pore in the endothelial cell plasma membrane. Brain endothelial cell damage leads to breakdown of the BBB (Popoff, 2011). In addition to its known effects on BBB vasculature, ETX has been found to specifically bind to and damage myelin when incubated with mammalian brain slices (Dorca-Arévalo et al., 2008; Linden et al., 2015; Wioland et al., 2015). This unique ability to interact specifically with the tissues that are damaged in MS, the BBB, and CNS myelin, makes it a promising candidate as an environmental MS trigger.

**Table 1:** ***Clostridium perfringens*** **toxinotypes, genotypes, and associated diseases**.

A	α	Humans: Gangrene, toxic enteritis, food poisoning, sporadic diarrhea, some cases of SIDS.
		Fowl: Necrotic enteritis.
		Foals, pigs: Diarrhea.
B	α, β, ε	Newborn Lambs: Dysentery.
		Newborn Calves and Foals: Hemorrhagic enteritis.
		Sheep and Goats: Enterotoxemia. Focal symmetric encephalomalacia.
C	α, β	Humans: Necrotic enteritis (Pigbel).
		Piglets, Lambs, Calves and Foals: Necrotic enteritis.
		Sheep: Enterotoxemia.
D	α, ε	Lambs, Sheep, Calves and Goats: Enterotoxemia. Focal symmetric encephalomalacia.
E	α, ι	Calves: Enterotoxemia.

Fingolimod was the first oral therapy to be approved for the treatment of MS. It was rationally engineered from the antifungal molecule Myriocin, which was later shown to possess immunosuppressive properties. Fingolimod and Myriocin are both structurally homologous to sphingosine, a lipid that is a necessary component of cell membrane sphingolipids (Strader et al., 2011). Similar to Myriocin, sphingosine is also known to possess antimicrobial properties. However, while Myriocin is antifungal in nature, sphingosine is antibacterial (Fischer et al., 2012). Interestingly, Fingolimod has been shown to mimic sphingosine's antibacterial properties by protecting the cystic fibrosis transmembrane conductance regulator (CFTR) knockout mouse from luminal airway infection by *Pseudomonas auerginosa* (Pewzner-Jung et al., 2014).

In the context of MS, Fingolimod is phosphorylated in the bloodstream and subsequently binds to the lymphocyte sphingosine-1-phosphate receptor 1 (S1PR1), causing rapid internalization of S1PR1. In the absence of surface S1PR1, lymphocytes are unable to egress from lymphoid tissues and cannot traffic to target tissues such as the brain; thus the rationale that Fingolimod may reduce the risk of MS relapse and the severity of attacks through immune modulation (Strader et al., 2011).

Teriflunomide is the active metabolite of the immunosuppressant Lenflunomide, which is currently approved for the treatment of rheumatoid arthritis (Munier-Lehmann et al., 2013). Teriflunomide inhibits *de novo* pyrimidine synthesis in rapidly dividing cells such as clonally expanding lymphocytes, potentially mitigating an autoimmune attack against myelin. More specifically, Teriflunomide non-competitively inhibits dihydroorotate dehydrogenase, an enzyme involved in the first step of de novo pyrimidine synthesis. Memory B cells and T cells remain unaffected by Teriflunomide as they divide more slowly and can synthesize DNA by utilizing the pyrimidine salvage pathway (Bar-Or et al., 2014). Interestingly, dihydroorotate dehydrogenase inhibitors have been shown to arrest the growth of unicellular organisms such as plasmodium falciparum presumably by inhibiting *de novo* pyrimidine synthesis (Pavadai et al., 2016).

DMF is a fumaric acid ester, which was originally investigated for use as an antimicrobial preservative^1^. It was first used therapeutically to treat psoriasis based on a hypothesis that psoriasis is caused by a defect in fumarate mediated carbohydrate metabolism in the skin. In the early 2000s, a German neurologist noticed that MS patients taking DMF for concurrent psoriasis showed stabilization of their MS symptoms and a reduction in relapse rates (Phillips and Fox, 2013).

DMF has been shown to react with thiol-containing molecules such as the cellular antioxidant, glutathione, and the cysteine residues of proteins via a chemical reaction called the Michael addition (Brennan et al., 2015). Although DMF initially depletes mammalian cells of glutathione, its proposed protective action in MS stems from its ability to alkylate key cysteine residues in the redox sensitive protein Kelch-Like ECH-Associated Protein 1 (Keap1). Keap1 normally inhibits Nuclear factor (erythroid-derived 2)-like 2 (Nrf2) from translocating to the nucleus and activating antioxidant gene expression. When the cysteine residues of Keap1 are oxidized by reactive oxygen species (ROS) or organic electrophiles such as DMF, Keap1 dissociates from Nrf-2, allowing nuclear translocation to occur. This elicits a robust antioxidant cellular response. The initial decrease in cellular glutathione after DMF treatment is followed by a sharp glutathione increase via the Nrf-2 pathway, which may protect vulnerable cells in MS (Phillips and Fox, 2013).

Although Fingolimod, Teriflunomide, and DMF have proposed mechanisms for how they protect the central nervous system from MS mediated damage, one unexplored possibility is that these orally administered agents may inhibit the growth of neurotoxin-secreting gut bacteria. Because, during log-phase growth, *C. perfringens* secretes ETX, a toxin that specifically targets the BBB and the myelin sheath, we chose to investigate the effect of these oral MS therapies on *C. perfringens* growth *in vitro*.

## Methods

### Drugs and compounds

All drugs and compounds used in this study were purchased from Sigma Aldrich.

### Bacterial strains and growth conditions

*C. perfringens* ATCC 13124 (type A), ATCC 3626 (type B), ATCC 51880 (type C), ATCC 3631 (type D), ATCC 27324 (type E), and two type A clinical isolates provided by New York Presbyterian Hospital were used for the initial screen while the “type strain,” ATCC 13124, was used for all subsequent experiments. Bacteria were cultured anaerobically at 37°C overnight using the GasPak 100 system (BD). Anaerobiosis was achieved by pre-reducing the culture media using an anaerobic jar containing a GasPak EZ Anaerobe System sachet for a minimum of 6 h before inoculation. After inoculation, the GasPak sachet was replaced for the overnight culture.

### Experimental procedures

The compounds used for the initial growth inhibition screen were diluted to a final concentration of 500 μg/ml in Mueller Hinton broth (BD) and media was inoculated with 5 × 10^6^ colony-forming units (CFUs) of different *C. perfringens* strains. Minimal Inhibitory Concentration values (≥95% growth inhibition, MIC_95_) were determined for inhibitory compounds using cation adjusted Mueller Hinton II broth (BD). Inhibitory compounds were diluted serially from 512 μg/ml down to 0.5 μg/ml, inoculated with 5 × 10^5^ CFUs of *C. perfringens* and then anaerobically cultured at 37°C overnight as previously described. Culture conditions for each compound were performed in triplicate and bacterial growth was determined by measuring OD_600_-values from 1 ml of re-suspended bacteria.

### Statistical analysis

Results are representative of data obtained from repeated independent experiments. Each value represents the mean ± *SD* for three replicates. Statistical analysis was performed using the two-tailed Student *t*-test (GraphPad Software, San Diego, CA, USA).

## Results

**Figure 1:**
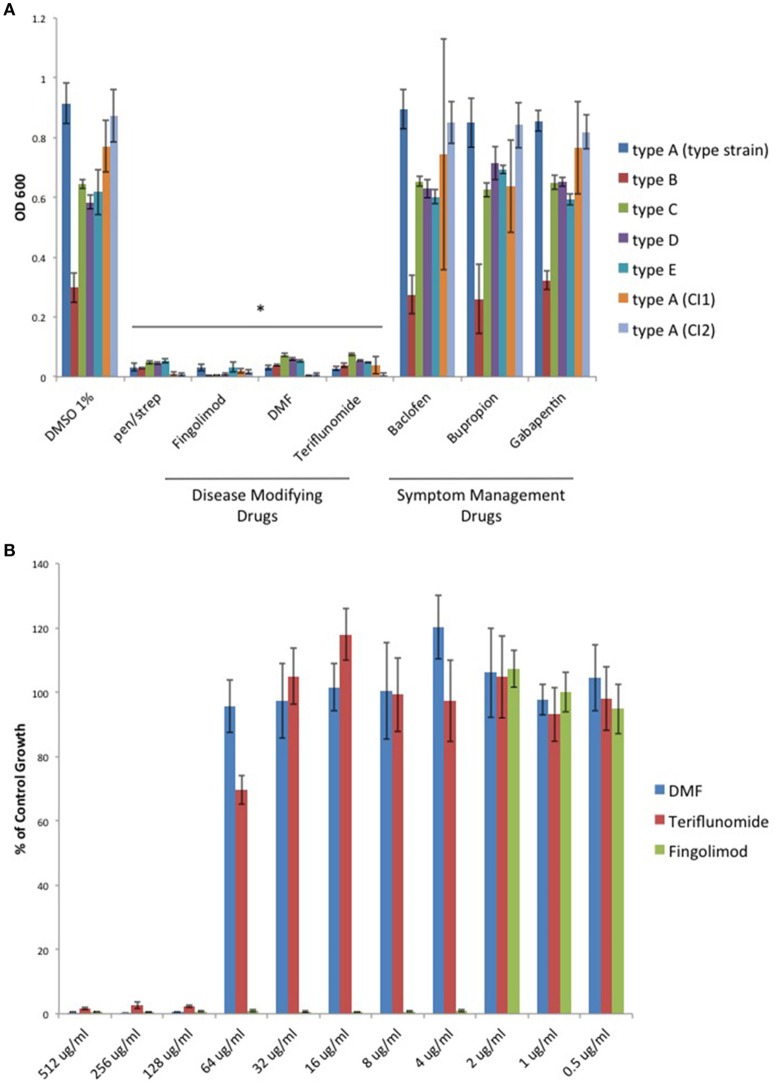
**Oral disease modifying MS drugs inhibit ***C. perfringens*** growth, while oral drugs used for MS symptom management do not. (A)** *C. perfringens* toxinotypes A, B, C, D, E, and two type A clinical isolates (CI1 and CI2) were anaerobically cultured in the presence of 500 μg/ml of oral DMDs, Fingolimod, DMF, and Teriflunomide, each of which significantly inhibited bacterial growth for all strains tested, similar to what was observed when bacteria were cultured in the presence of known antibiotic mixture penicillin/streptomycin (pen/strep, 100 U/ml). In contrast, oral SMDs failed to inhibit *C. perfringens* growth, yielding OD_600_-values similar to that of the DMSO vehicle control. Data are presented as means from three independent experiments. Error bars represent standard deviations, and asterisks indicate that results are statistically significant compared with the DMSO vehicle controls (Student's *t*-test, ^*^P < 0.001). **(B)** Serial dilutions of inhibitory oral DMDs were performed and the type strain, *C. perfringens* ATCC 13124 (type A), was cultured in each condition. OD_600_-values for each concentration were divided by that of the corresponding dilution for the DMSO vehicle control. MIC-values were plotted for each oral DMD revealing that Fingolimod was the most potent compound with an MIC_95_ of 4 μg/ml, compared to 128 μg/ml for DMF and Teriflunomide.

With renewed interest in gut bacteria and their potential involvement in the pathogenesis of MS (Bhargava and Mowry, 2014), we wished to determine if oral disease-modifying drugs (DMDs) have the ability to modulate growth of *C. perfringens* since type B and D strains secrete ETX during log-phase growth. Therefore, we tested if oral DMDs affected the growth of *C. perfringens* toxinotypes A–E. We compared the growth inhibitory effects of Fingolimod, DMF, and Teriflunomide to that of oral symptom management drugs (SMDs) Baclofen, Bupropion, and Gabapentin; drugs not thought to alter the disease course of MS. We exposed *C. perfringens* cultures to 500 μg/ml of each compound, allowed for overnight anaerobic growth, and determined the optical density (OD_600_) the following day. We found that each oral DMD significantly inhibited all *C. perfringens* toxinotypes and strains tested, while the oral SMDs did not (Figure 1A). We then plotted the minimal inhibitory concentration (MIC) values for each inhibitory compound and found that Fingolimod was the most potent inhibitor at 4 μg/ml (Figure 1B).

**Figure 2:**
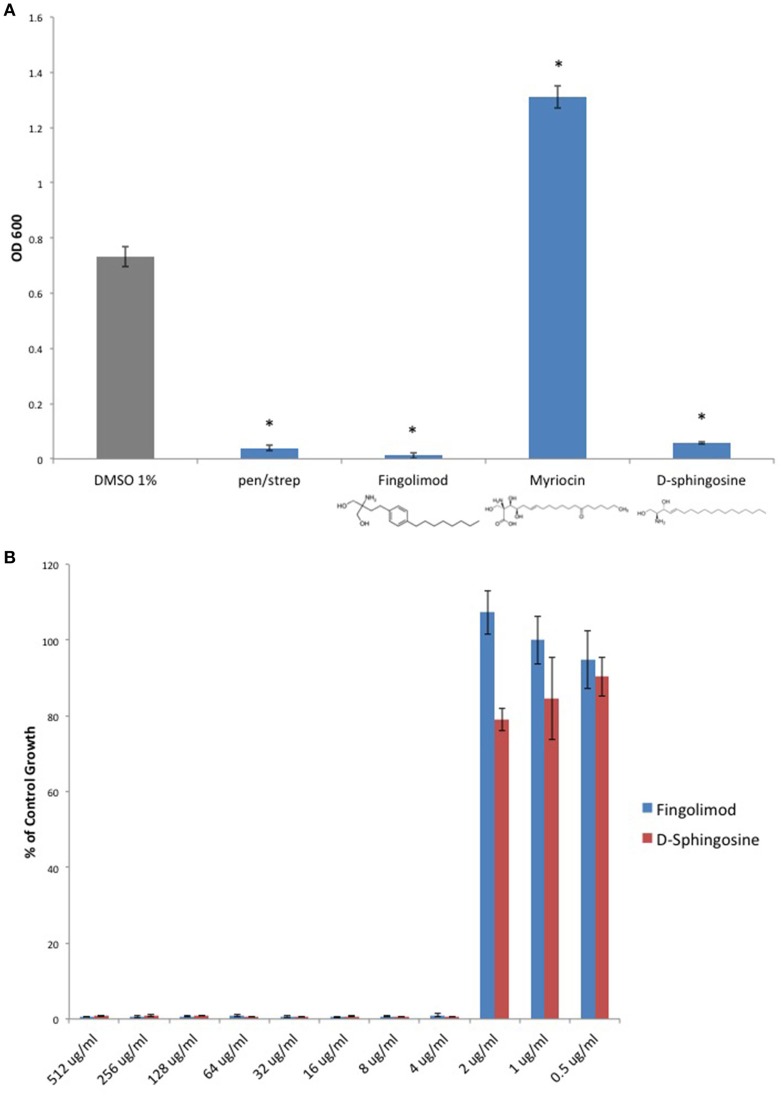
**D-sphingosine, a Fingolimod related compound, inhibits ***C. perfringens*** growth. (A)** Type strain, *C. perfringens* ATCC 13124, was anaerobically cultured in the presence of 500 μg/ml Fingolimod and other sphingoid compounds, D-sphingosine and Myriocin. Like Fingolimod, D-sphingosine also inhibited *C. perfringens* growth. However, Myriocin failed to inhibit the bacterium. Instead, Myriocin enhanced *C. perfringens* growth above that of the DMSO vehicle control. Data are presented as means from three independent experiments. Error bars represent standard deviations, and asterisks indicate that results are statistically significant compared with the DMSO vehicle control (gray); Student's *t*-test, ^*^P < 0.0001. **(B)** Serial dilutions of inhibitory sphingoid molecules were performed and *C. perfringens* ATCC 13124 was cultured at each dilution. OD_600_-values for each dilution were divided by that of the corresponding DMSO vehicle control dilution. MIC-values were plotted for each of the inhibitory sphingoid compounds revealing that D-sphingosine mimics Fingolimod's antibacterial potency with an MIC_95_-value of 4 μg/ml.

Because Fingolimod is a homolog of D-sphingosine and Myriocin, both of which have been shown to possess antimicrobial properties (Fischer et al., 2012), we compared the inhibitory activity of Fingolimod to these related sphingoid molecules. We exposed the type strain, *C. perfringens* ATCC 13124 (type A), to Fingolimod, D-sphingosine, and Myriocin and identified that, like Fingolimod, D-sphingosine also displayed inhibitory activity. Myriocin failed to inhibit *C. perfringens*, but instead, enhanced bacterial growth (Figure 2A). We then plotted and compared MICs for Fingolimod and D-sphingosine and determined that D-sphingosine displayed a similar inhibitory potency to that of Fingolimod with an MIC_95_ of 4 μg/ml (Figure 2B).

Although an antioxidant mechanism has been proposed for how DMF protects cells against MS mediated damage, DMF was originally investigated for use as an antimicrobial compound^1^. Interestingly, DMF was also found to inhibit the growth of *Clostridium botulinum* (Dymicky et al., 1987), a bacterial species closely related to *C. perfringens*. DMF is known to be a Michael acceptor and its ability to affect the redox status of cells stems from its electrophilic nature. Michael acceptors accept electrons during the Michael reaction, while nucleophilic thiols (Michael donors) donate electrons. The Michael reaction results in covalent alkylation of the sulfhydryl group. This covalent linkage permanently inactivates thiol-containing molecules if the thiol is necessary for the molecule's function, as is the case for glutathione and its antioxidant properties (Brennan et al., 2015).

**Figure 3:**
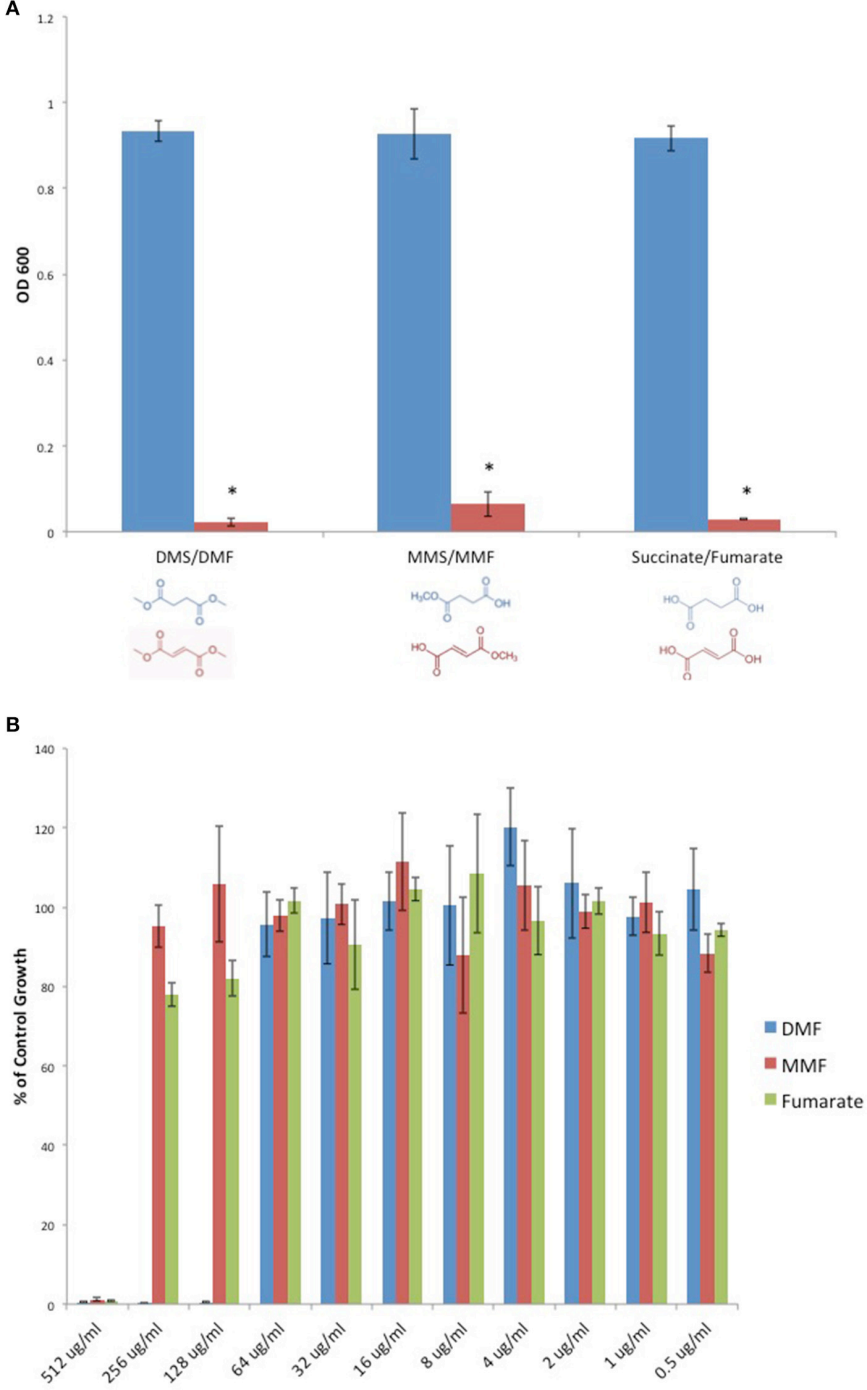
**Fumarates inhibit ***C. perfringens*** growth while their related saturated succinates do not. (A)** The anaerobic growth of *C. perfringens* ATCC 13124 was compared for DMF and its corresponding succinate (DMS), MMF and its corresponding succinate MMS, fumarate, and succinate. In each case, the unsaturated fumarate compounds (red) displayed inhibitory activity, while the saturated succinates (blue) did not. Data are presented as means from three independent experiments. Error bars represent standard deviations, and asterisks indicate that the inhibition of bacterial growth observed in the presence of unsaturated fumarates is statistically significant when compared to the bacterial growth observed in the presence of corresponding saturated succinates; Student's *t*-test, ^*^P < 0.0001). **(B)** Serial dilutions of inhibitory fumarate compounds were performed and *C. perfringens* ATCC 13124 was cultured at each dilution. OD_600_-values for each dilution were divided by that of the corresponding DMSO vehicle control dilution. MIC-values were plotted for each of the inhibitory fumarates revealing that DMF is approximately four times more potent that MMF and fumarate.

We sought to determine if DMF's antimicrobial activity extended to *C. perfringens*. Furthermore, we examined DMF's Michael acceptor activity as pertaining to its antimicrobial properties. We screened DMF and its metabolites, monomethyl fumarate (MMF), and fumarate and found that each compound inhibited the growth of type strain, *C. perfringens* ATCC 13124. However, their saturated succinate counterparts dimethyl succinate (DMS), monomethyl succinate (MMS), and succinate, molecules devoid of Michael acceptor activity due to reduction of the α, β carbon double bond, failed to inhibit *C. perfringens* (Figure 3A). We plotted MIC-values for DMF, MMF, and fumarate and found that DMF was four times more potent than either MMF or fumarate (Figure 3B).

**Figure 4:**
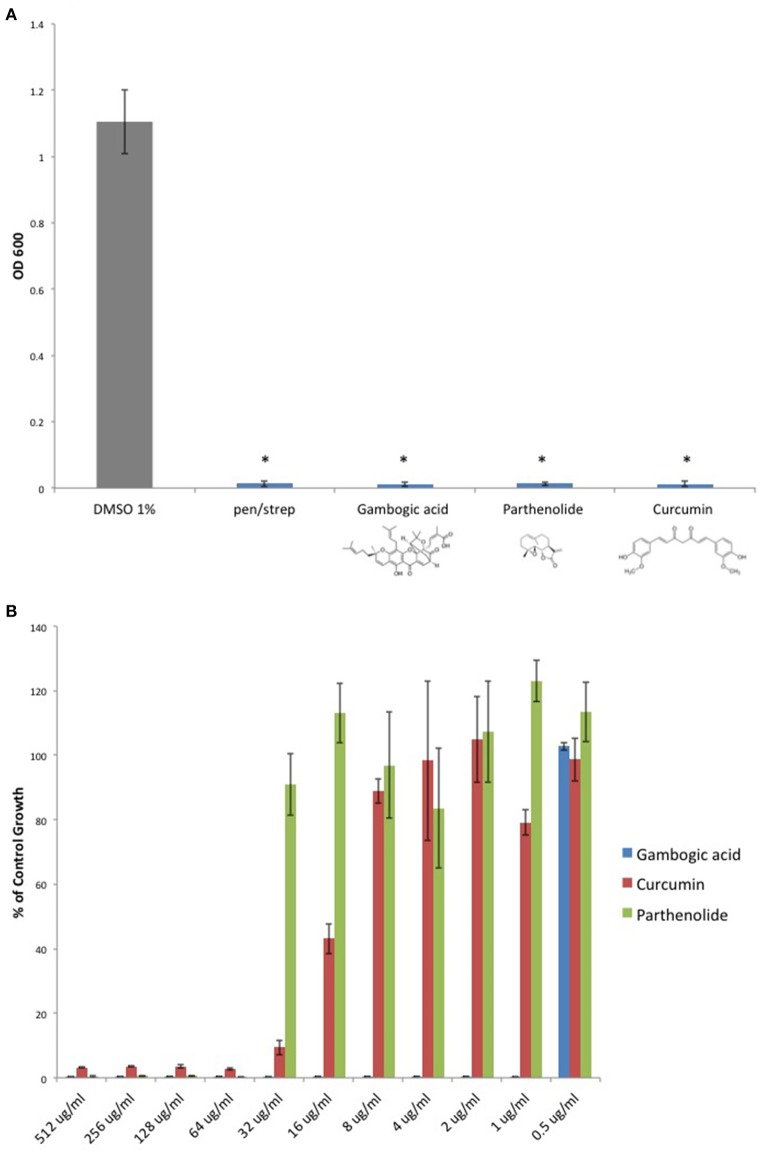
**Natural product Michael acceptors inhibit ***C. perfringens*** growth. (A)** Plant derived Michael acceptors were tested for inhibitory activity against *C. perfringens* ATCC 13124. Bacteria were grown anaerobically in the presence of 500 μg/ml Gambogic acid, Parthenolide, and Curcumin. Each natural product Michael acceptor successfully inhibited *C. perfringens* growth, similar to what was observed when bacteria were cultured in the presence of known antibiotic penicillin/streptomycin (pen/strep, 100 U/ml). Data are presented as means from three independent experiments. Error bars represent standard deviations, and asterisks indicate that results are statistically significant compared with the DMSO vehicle control (gray); Student's *t*-test, ^*^P < 0.0001. **(B)** Serial dilutions of inhibitory natural product Michael acceptors were performed and *C. perfringens* ATCC 13124 was cultured at each dilution. OD_600_-values for each dilution were divided by that of the corresponding DMSO vehicle control dilution. MIC-values were plotted for each compound revealing Gambogic acid as the most potent with an MIC_95_ of 1 μg/ml when compared to Parthenolide and Curcumin, which each inhibit *C. perfringens* at 64 μg/ml.

Given that Michael acceptor activity was necessary for *C. perfringens* inhibition by DMF and its fumarate metabolites, we sought to determine if unrelated molecules that share the α, β unsaturated carbonyl structure could also inhibit *C. perfringens*. We screened Michael acceptors from a diverse group of chemical families and found that natural product Michael acceptors Gambogic acid (a xanthonoid), Parthenolide (a sesquiterpenoid), and Curcumin (a curcuminoid) each inhibited *C. perfringens* (Figure 4A). Interestingly, we found that Gambogic acid was particularly inhibitory with an MIC_95_ of 1 μg/ml (Figure 4B).

**Figure 5:**
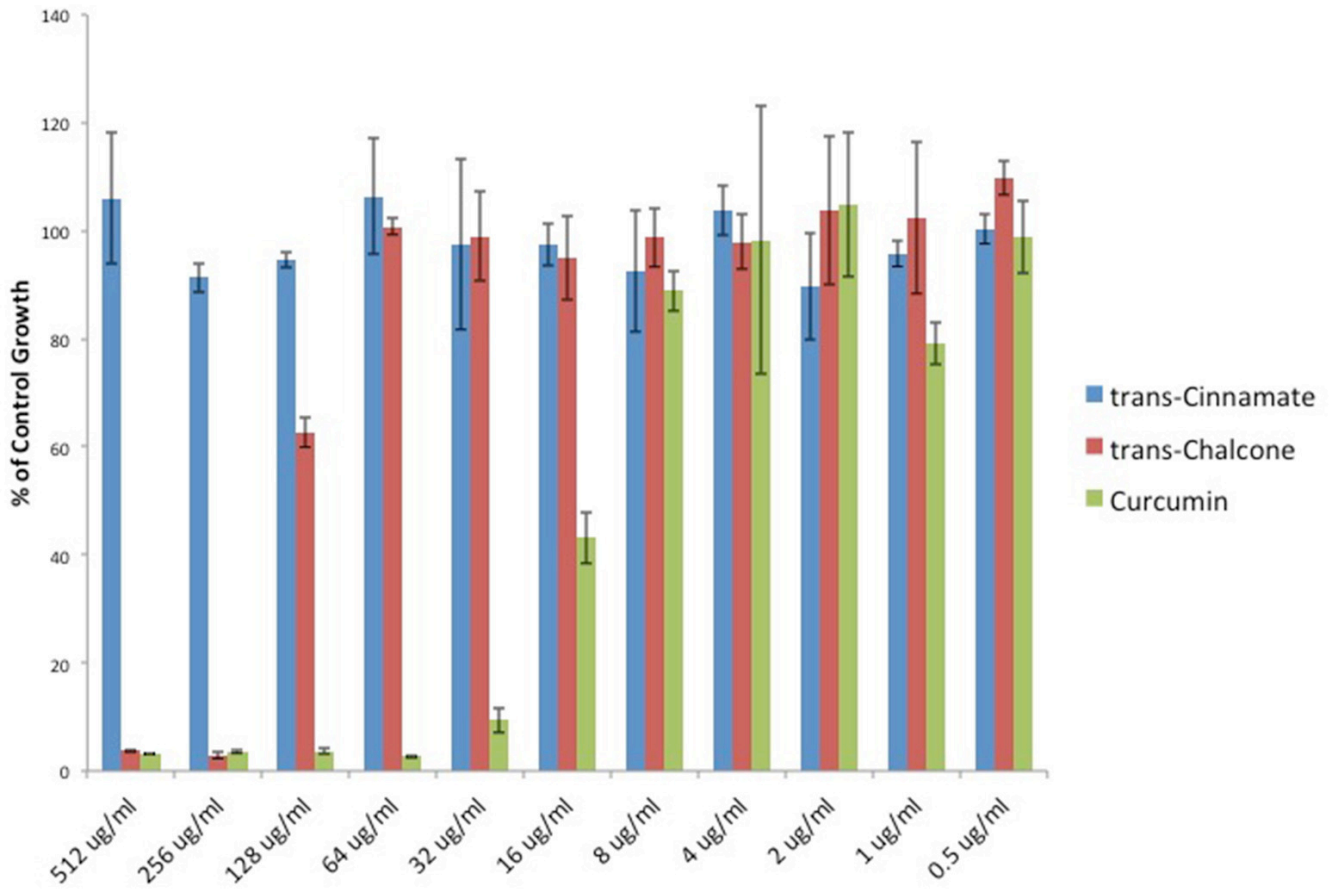
**Michael acceptor ***C. perfringens*** growth inhibition reflects known reactivity potencies**. Serial dilutions of α, β unsaturated carbonyls were performed and *C. perfringens* ATCC 13124 was cultured at each dilution. OD_600_-values for each dilution were divided by that of the corresponding DMSO vehicle control dilution. MIC-values were plotted for each compound revealing that Cinnamic acid displays no inhibitory activity, while Curcumin is four times more potent than trans-Chalcone. The relative inhibitory potencies of each compound corresponds almost exactly with the relative Michael acceptor potencies as demonstrated by Dinkova-Kostova et al. (2001).

To provide additional evidence that Michael acceptor activity is indeed critical to the antibacterial properties of α, β unsaturated carbonyls, we searched the literature to find compounds for which experimental values of Michael reaction potencies have been determined. Dinkova-Kostova et al. determined the potencies of several plant derived Michael acceptors for their ability to induce cellular quinone reductase activity; a cellular marker for a compound's reactivity with sulfhydryl containing molecules (Dinkova-Kostova et al., 2001). In our study, the antibacterial potencies of Cinnamic acid, trans-Chalcone, and Curcumin mirrored the Michael acceptor potencies described by Dinkova-Kostova and colleagues (Figure 5). Cinnamic acid was previously shown to be inactive as a Michael acceptor, and in our hands, this compound failed to inhibit *C. perfringens*. Furthermore, Curcumin was found to be approximately four times more potent than trans-Chalcone (ratio = 4.13; Dinkova-Kostova et al., 2001). Likewise, we found that Curcumin was four times more potent than trans-Chalcone as a *C. perfringens* inhibitor (ratio = 4).

**Figure 6:**
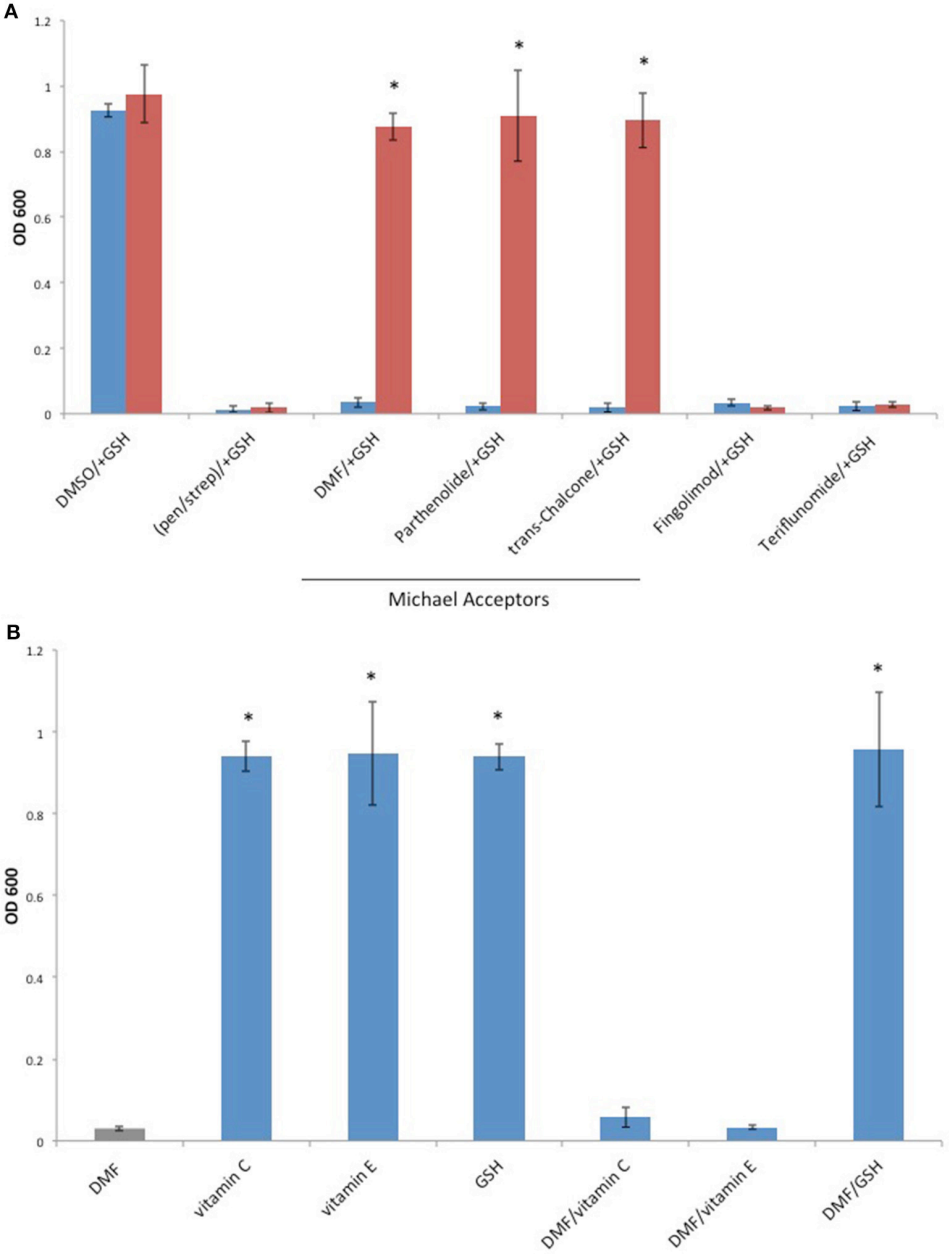
**The Michael donor, glutathione (GSH), abolishes Michael acceptor inhibition of ***C. perfringens*** growth. (A)** *C. perfringens* ATCC 13124 was cultured anaerobically at a Michael acceptor (DMF, Parthenolide, and trans-Chalcone), and non-Michael acceptor (Fingolimod and Teriflunomide) concentration of 500 μg/ml, with (red) or without (blue) the addition of an equal quantity of exogenous Michael donor, GSH. Only Michael acceptor mediated growth inhibition could be abolished by the addition of exogenous GSH. The inhibitory activity Fingolimod and Teriflunomide remained unaffected by the presence of GSH, similar to what was observed when bacteria were cultured in the presence of pen/strep and GSH. Data are presented as means from three independent experiments. Error bars represent standard deviations, and asterisks indicate that GSH aided growth recovery is statistically significant when compared to the lack of growth recovery in the absence of GSH; Student's *t*-test, ^*^P < 0.001. **(B)** *C. perfringens* ATCC 13124 was cultured in the presence of DMF, vitamin C, vitamin E, and GSH each at concentration of 250 μg/ml. Only the Michael acceptor, DMF, inhibited bacterial growth. DMF was then paired with the antioxidants, vitamin C, vitamin E, and GSH at concentrations of 250 μg/ml for each compound. The Michael donor antioxidant, GSH, abolished DMF inhibition. However, the non-Michael donor antioxidants, vitamin C and vitamin E, were unable to abolish DMF's inhibitory effect on *C. perfringens* growth. Data are presented as means from three independent experiments. Error bars represent standard deviations, and asterisks indicate that results are statistically significant compared with the DMF control (gray); Student's *t*-test, ^*^P < 0.001.

Since Michael acceptors react with thiols and deplete cellular glutathione levels (Brennan et al., 2015), we surmised that Michael acceptor inhibition of *C. perfringens* might be abolished by the addition of exogenous glutathione. To test this, we compared *C. perfringens* growth in the presence of Michael acceptors with and without an equal quantity of exogenous glutathione. We also tested the effect of glutathione on the inhibitory activity of each of the oral MS DMDs. Glutathione completely abolished growth inhibition by the known Michael acceptors in our study, but failed to abolish the inhibitory effects of Fingolimod and Teriflunomide (Figure 6A). Because glutathione is an antioxidant, as are vitamins C and E, we sought to determine if the glutathione's abrogation of Michael acceptor antibacterial activity is based on its nucleophilic behavior or due, more generally, to its antioxidant properties. *C. perfringens* was challenged with DMF in the presence of vitamin C, vitamin E, or the Michael donor, glutathione. Of the antioxidant panel, only the Michael donor, glutathione, was able to neutralize DMF's inhibitory effect (Figure 6B).

## Discussion

In this study, we have shown that each of the oral DMDs approved for the treatment of MS, Fingolimod, Teriflunomide, and DMF, inhibits the *in vitro* growth of the epsilon toxin-secreting gut bacterium, *C. perfringens*. In contrast, oral therapies used specifically for symptomatic management fail to prevent *C. perfringens* growth. Of note, Fingolimod proved to be bactericidal, while Teriflunomide and DMF were bacteriostatic (Supplemental Figure 1). The antibacterial properties of oral DMDs raises the possibility that modulation of the intestinal microbiota may play a role in the clinical efficacy of these compounds. Preventing *C. perfringens* growth and toxin production may serve as a specific example of this. Furthermore, we have identified two distinct classes of molecules capable of inhibiting *C. perfringens*; namely sphingoid compounds such as Fingolimod and D-sphingosine, and Michael acceptors such as DMF, its fumarate metabolites, and various natural products that are α, β unsaturated carbonyls.

Important factors must be considered when attempting to extrapolate these *in vitro* findings to what may be occurring in the human gut. First, how do the *in vitro* inhibitory concentrations compare to concentrations found in the human gut? The resting volume of the human stomach is ~0.08 L (Johnson, 1994), which yields a calculated gut concentration of 6.3 μg/ml for Fingolimod (MIC_95_ = 4 μg/ml), 87–175 μg/ml for Teriflunomide (MIC_95_ = 128 μg/ml), and 1500–3000 μg/ml for DMF (MIC_95_ = 128 μg/ml). Therefore, each compound's MIC_95_ is within the calculated range of the therapeutic concentration that will enter the small intestine. Furthermore, DMF is a delayed released capsule that dissolves in the more basic pH of the small intestine (Gold et al., 2016). Local release of DMF may increase its concentration in the small intestine where *C. perfringens* resides.

Second, our experimental growth conditions are likely to be more favorable to *C. perfringens* growth than the intestinal milieu. Anexic, *in vitro* growth protects *C. perfringens* from competition with other bacteria for nutrients. In addition, *C. perfringens* will not be exposed to toxic molecules secreted by competing bacteria such as bacteriocins or host derived antibacterial molecules such as defensins. Therefore, the MIC_95_ for each of the oral DMDs may be considerably less in an environment such as the human intestine where *C. perfringens* must contend with a multitude of external factors.

Conversely, each of the oral DMDs possesses a significant degree of hydrophobicity, and lipid-binding molecules within the gut lumen may sequester these compounds, preventing toxic interactions with gut bacteria. Specifically considering DMF, a Michael acceptor, extracellular nucleophiles present in the gut may react with its electrophilic β carbon before it can enter the bacterial cell, possibly diminishing its antibacterial activity within the gut.

DMF's Michael reaction-dependent inhibition of *C. perfringens* growth may be explained by its ability to deplete this bacterium of thiol containing compounds. It is striking that nucleophilic thiols not only play an important role in mammalian cell homeostasis, but are also necessary substrates for *C. perfringens* growth. This bacterium depends on an organic source of sulfur (thiols) and will not grow with strictly inorganic sources (${\text{SO}}_{4}^{2-}$, ${\text{SO}}_{3}^{2-}$, S_2_O_3_^2−^, and S_i_; Fuchs and Bonde, 1957). Therefore, depleting *C. perfringens* of thiols may contribute to Michael acceptor mediated growth inhibition.

Although Teriflunomide is an α, β unsaturated carbonyl, we have shown that glutathione has no effect on its ability to inhibit *C. perfringens* growth. It is tempting to speculate that Teriflunomide inhibits *de novo* pyrimidine synthesis in rapidly dividing bacterial cells, as it does in mammalian cells, via inhibition of dihydroorotate dehydrogenase; a gene that has been annotated for *C. perfringens* in the Uniprot Knowledgebase. However, we have not examined the inhibitory mechanism of Teriflunomide in the present study.

That Michael acceptors such as DMF and its fumarate metabolites inhibit *C. perfringens* may open the door to development of new oral MS therapies derived from the Michael acceptor functional class. Gambogic acid has been used in Eastern medicine for centuries to treat intestinal ailments and parasites (Wu et al., 2004), and in our hands, it displays an impressive antibacterial potency (MIC_95_ = 1 μg/ml).

We searched for Michael acceptors currently approved for human use that possess no known immunosuppressive properties. The naphthoquinone, Menadione (vitamin K3), is a synthetic precursor for vitamin K. It is commonly used as a dietary supplement for livestock and as a cost effective vitamin K replacement therapy in developing countries. Of note, Menadione has recently been shown to inhibit *S. aureus* and *B. anthracis* growth, and to suppress *S. aureus* secretion of toxic shock syndrome toxin 1 (TSST-1; Schlievert et al., 2013). Similarly, we find that Menadione inhibits *C. perfringens* growth, but related compounds with long aliphatic side chains, vitamin K1, vitamin K2, and ubiquinone do not (Supplemental Figure 2A). While Menadione's MIC95-value was found to be 64 μg/ml (Supplemental Figure 2B), the inactivity of the Menadione related compounds, all of which are electron carriers in the electron transport chain, might be due to the fact that they are sequestered in the cell membrane by their aliphatic side chains. Membrane sequestration may protect cytosolic nucleophiles from undergoing Michael addition and subsequent depletion. Additionally, unlike Menadione but similar to Teriflunomide, these molecules possess a third σ bond at the β carbon position. This may prevent nucleophilic attack due to steric hindrance and abolish Michael acceptor activity (Schwöbel et al., 2010).

In light of the serious side effects associated with current oral DMDs, this study may be of immediate clinical importance. Some of these adverse effects are due to immunosuppression of the CNS, as evidenced by increased risk of JC virus infection and progressive multifocal leukoencephalopathy (PML, FDA Drug Safety Communication, 2014; Brooks, 2015). Perhaps new antibacterial compounds based on these early oral DMDs, but lacking their immunosuppressive properties, may be of use in treating MS. For example, Fingolimod/D-sphingosine related compounds lacking hydroxyl head groups will not undergo phosphorylation and will not target lymphocyte S1PR1. Such compounds would not be immunosuppressive and may reduce the risk of JC virus infection and the development of PML. Along these lines, we have tabulated the MIC95-values for each inhibitory compound used in this study (Table 2).

**Table 2:** **Minimal Inhibitor Concentrations for all inhibitory compounds used in the study**.

**Compound**	**MIC_95_ (μg/ml)**
Gambogic Acid	1
Fingolimod	4
D-sphingosine	4
Curcumin	64
Parthenolide	64
Menadione (vitamin K3)	64
Teriflunomide	128
Dimethyl fumarate (DMF)	128
Trans-Chalcone	256
Monomethyl fumarate (MMF)	512
Fumarate	512

## Author contributions

KR conceived the study. KR, VF, and TV designed the study. KR, VF, and TV performed the literature search. KR collected the data and wrote the paper. All authors analyzed the data.

## Funding

This work was generously supported by the Rockefeller University's Robertson Therapeutic Development Fund (RTDF), the Center for Disorders of the Digestive System (CDDS), and the Weill Cornell Tisch Family Research Fund.

### Conflict of interest statement

All authors are named as inventors on a pending patent entitled, “Methods to protect against and treat multiple sclerosis,” (Publication number CA2899961 A1), which identifies *Clostridium perfringens* epsilon toxin as candidate trigger for multiple sclerosis.

## Footnotes

^1^Pest control. Patent Publication Number: US2218181 A.

## Supplementary material

Supplementary Figure 1 **Fingolimod is bactericidal, while DMF and Teriflunomide are bacteriostatic**. *C. perfringens* ATCC 13124 was anaerobically cultured to stationary phase and exposed to Fingolimod (500 μg/ml), DMF (500 μg/ml), Teriflunomide (500 μg/ml), pen/strep (100 U/ml) or DMSO vehicle control for 4 h under anaerobic conditions. Treated cultures were diluted 1000 fold in fresh, pre-reduced Mueller Hinton broth and cultured anaerobically. Fingolimod inhibited *C. perfringens* growth in a bactericidal fashion, similar to what was observed with the known bactericidal antibiotic mixture pen/strep, as post-treatment dilution and repeat culture failed to restore bacterial growth. Conversely, DMF and Teriflunomide were shown to be bacteriostatic, as post-treatment dilution and culture successfully restored bacterial growth. Data are presented as means from three independent experiments. Error bars represent standard deviations, and asterisks indicate that results are statistically significant compared with the DMSO vehicle control (gray); Student's *t*-test, ^*^*P* < 0.0001.

Supplementary Figure 2 **Synthetic vitamin K3, Menadione, inhibits *C. perfringens* growth. (A)** *C. perfringens* ATCC 13124 was anaerobically cultured in the presence of vitamin K homologs, vitamins K1, K2, K3, and ubiquinone. Only the synthetic vitamin K3 (Menadione) inhibited bacterial growth, similar to what was observed when bacteria were cultured in the presence known antibiotic penicillin/streptomycin (pen/strep, 100 U/ml). Conversely, bacteria derived vitamin K2 enhanced *C. perfringens* growth, while plant derived vitamin K1 and mammalian ubiquinone yielded OD_600_-values similar to that of the DMSO vehicle control. Data are presented as means from three independent experiments. Error bars represent standard deviations, and asterisks indicate that results are statistically significant compared with the DMSO vehicle control (gray); Student's *t*-test, ^*^*P* < 0.001. **(B)** Serial dilutions of Menadione were performed and *C. perfringens* ATCC 13124 was cultured at each dilution. OD_600_-values for each dilution were divided by that of the corresponding DMSO vehicle control dilution. MIC-values were plotted yielding an MIC_95_-value of 64 μg/ml.
