# Supplementary figures and images for: Oral Multiple Sclerosis Drugs Inhibit the In vitro Growth of Epsilon Toxin Producing Gut Bacterium, Clostridium perfringens
Source: Front Cell Infect Microbiol. 2017 Jan 25;7:11. doi: 10.3389/fcimb.2017.00011 (PMC5263136; doi:10.3389/fcimb.2017.00011)

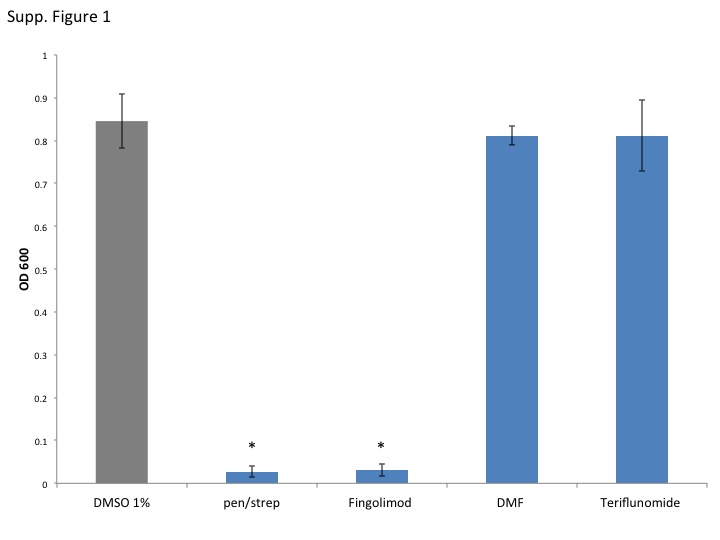

Supplement: Supplementary Figure 1 — Fingolimod is bactericidal, while DMF and Teriflunomide are bacteriostatic. C. perfringens ATCC 13124 was anaerobically cultured to stationary phase and exposed to Fingolimod (500 μg/ml), DMF (500 μg/ml), Teriflunomide (500 μg/ml), pen/strep (100 U/ml) or DMSO vehicle control for 4 h under anaerobic conditions. Treated cultures were diluted 1000 fold in fresh, pre-reduced Mueller Hinton broth and cultured anaerobically. Fingolimod inhibited C. perfringens growth in a bactericidal fashion, similar to what was observed with the known bactericidal antibiotic mixture pen/strep, as post-treatment dilution and repeat culture failed to restore bacterial growth. Conversely, DMF and Teriflunomide were shown to be bacteriostatic, as post-treatment dilution and culture successfully restored bacterial growth. Data are presented as means from three independent experiments. Error bars represent standard deviations, and asterisks indicate that results are statistically significant compared with the DMSO vehicle control (gray); Student's t-test, *P < 0.0001. [file Image1.jpeg]

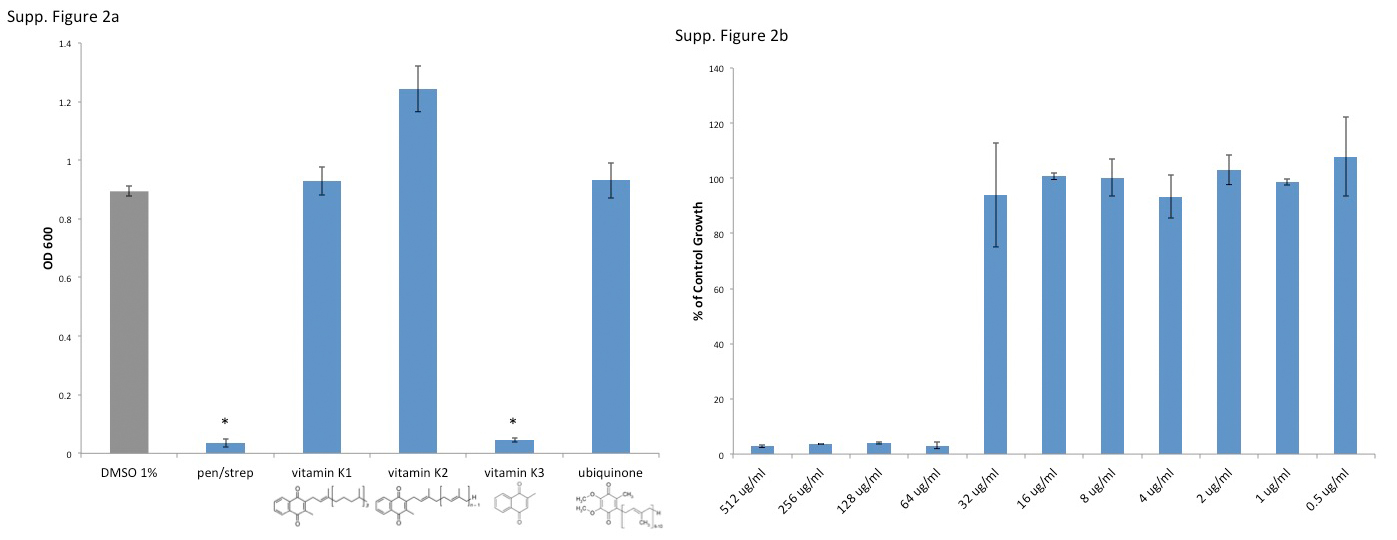

Supplement: Supplementary Figure 2 — Synthetic vitamin K3, Menadione, inhibits C. perfringens growth. (A) C. perfringens ATCC 13124 was anaerobically cultured in the presence of vitamin K homologs, vitamins K1, K2, K3, and ubiquinone. Only the synthetic vitamin K3 (Menadione) inhibited bacterial growth, similar to what was observed when bacteria were cultured in the presence known antibiotic penicillin/streptomycin (pen/strep, 100 U/ml). Conversely, bacteria derived vitamin K2 enhanced C. perfringens growth, while plant derived vitamin K1 and mammalian ubiquinone yielded OD600-values similar to that of the DMSO vehicle control. Data are presented as means from three independent experiments. Error bars represent standard deviations, and asterisks indicate that results are statistically significant compared with the DMSO vehicle control (gray); Student's t-test, *P < 0.001. (B) Serial dilutions of Menadione were performed and C. perfringens ATCC 13124 was cultured at each dilution. OD600-values for each dilution were divided by that of the corresponding DMSO vehicle control dilution. MIC-values were plotted yielding an MIC95-value of 64 μg/ml. [file Image2.jpg]
